# Removal of aromatic inhibitors produced from lignocellulosic hydrolysates by *Acinetobacter baylyi* ADP1 with formation of ethanol by *Kluyveromyces marxianus*

**DOI:** 10.1186/s13068-019-1434-7

**Published:** 2019-04-23

**Authors:** Anita Singh, Stacy R. Bedore, Nilesh K. Sharma, Sarah A. Lee, Mark A. Eiteman, Ellen L. Neidle

**Affiliations:** 1 Department of Environmental Sciences, Central University of Jammu, Rahya-Suchani, Bagla, India; 2 School of Chemical, Materials and Biomedical Engineering, University of Georgia, Athens, GA 30602 USA; 3 Department of Microbiology, University of Georgia, Athens, GA 30602 USA

**Keywords:** *Acinetobacter baylyi*, Detoxification, Ethanol, *Kluyveromyces marxianus*, Microbial consortium

## Abstract

**Background:**

Lignocellulosic biomass is an attractive, inexpensive source of potentially fermentable sugars. However, hydrolysis of lignocellulose results in a complex mixture containing microbial inhibitors at variable composition. A single microbial species is unable to detoxify or even tolerate these non-sugar components while converting the sugar mixtures effectively to a product of interest. Often multiple substrates are metabolized sequentially because of microbial regulatory mechanisms. To overcome these problems, we engineered strains of *Acinetobacter baylyi* ADP1 to comprise a consortium able to degrade benzoate and 4-hydroxybenzoate simultaneously under batch and continuous conditions in the presence of sugars. We furthermore used a thermotolerant yeast, *Kluyveromyces marxianus*, to convert the glucose remaining after detoxification to ethanol.

**Results:**

The two engineered strains, one unable to metabolize benzoate and another unable to metabolize 4-hydroxybenzoate, when grown together removed these two inhibitors simultaneously under batch conditions. Under continuous conditions, a single strain with a deletion in the *gcd* gene metabolized both inhibitors in the presence of sugars. After this batch detoxification using ADP1-derived mutants, *K. marxianus* generated 36.6 g/L ethanol.

**Conclusions:**

We demonstrated approaches for the simultaneous removal of two aromatic inhibitors from a simulated lignocellulosic hydrolysate. A two-stage batch process converted the residual sugar into a non-growth-associated product, ethanol. Such a two-stage process with bacteria (*A. baylyi)* and yeast (*K. marxianus*) is advantageous, because the yeast fermentation occurs at a higher temperature which prevents growth and ethanol consumption of *A. baylyi.* Conceptually, the process can be extended to other inhibitors or sugars found in real hydrolysates. That is, additional strains which degrade components of lignocellulosic hydrolysates could be made substrate-selective and targeted for use with specific complex mixtures found in a hydrolysate.

**Electronic supplementary material:**

The online version of this article (10.1186/s13068-019-1434-7) contains supplementary material, which is available to authorized users.

## Introduction

Lignocellulosic biomass represents an underutilized renewable resource readily available from forestry and agricultural residues. For example, about 60% of United States logging residue and forest thinning materials can be harvested, totaling 75 million tons annually [52]. Corn is the most widely planted agricultural crop in the U.S., and estimated 40 million tons of corn stover are sustainably available for bio-based products [20]. Promising “energy crops” have also been identified, such as switchgrass, Napier grass and *Miscanthus* that provide high yield/energy content require low maintenance, often grow on marginal lands, and do not divert food to fuel. The annual global production rate of potentially accessible primary biomass is 8–20 × 10^9^ tons [39], and this material is of interest for the production of liquid transportation fuels through chemical [16] or biological routes [12]. Estimated 400–600 billion liters of ethanol could be generated from these lignocellulosic crops, residues, and waste [23, 36, 41]. Agricultural residues from corn, wheat, rice, and sugarcane crops are particularly appealing, since they are distributed throughout the populous world.

Biomass is an attractive feedstock for the biochemical production of fuels and chemicals using a microbial conversion platform. However, such conversions encounter two significant challenges. First, although the large available volume and cost of biomass align well with the production of commodity chemicals such as ethanol, the variability of these feedstocks is problematic for microbial metabolism. Even a single feedstock can vary substantially: for example, in one study, corn stover contained 0.9–2.9% acetyl groups, 26–38% glucans, and 15–23% xylans on a whole biomass basis [47]. Similarly, cotton gin residue varied between 3 and 13% xylans and 20–38% glucans, with the composition dependent on the day of collection [1]. A microbial process ideally should be able to tolerate the inherent variability of the biomass feedstock.

The second major challenge for bioconversion processes is that methods used to liberate sugars involve high heat and pressure with acids/bases that generate microbial inhibitors such as acetic acid [25, 31, 38, 48], furans [14], and aromatic compounds [28, 49, 50]. The concentrations of these compounds depend on the biomass feedstock and the hydrolysis process. For example, using 4% phosphoric acid to hydrolyze sugarcane bagasse at 122 °C generated 3 g/L acetic acid and 0.6 g/L furfural [11], while hydrolysis of corn stover using dilute sulfuric acid with a short residence time and 200 °C generated 14 g/L acetic acid and 5 g/L furfural [6]. Thus, in addition to the inherent variability of sugar in the feedstocks, pre-treatment methods generate variation in the inhibitory mixtures. Despite progress, many difficulties remain in the microbial conversion of lignocellulose to fuels and chemicals, in particular the presence of the numerous inhibitors in hydrolysates and their variable composition [3, 45].

A single microbial species has not been identified that can detoxify or even tolerate the non-sugar components of lignocellulose hydrolysates while effectively converting the sugar mixtures to a product of interest. An alternative to the use of a single microbe is the use of a microbial consortium for detoxification and biochemical conversion of the sugars [9]. For example, the sugars glucose, xylose, and arabinose as well as the inhibitor acetic acid can be degraded simultaneously by a consortium of different *Escherichia coli* strains [55]. As an extension of this approach, inhibitors could be removed in a first stage, followed by a second process targeting bioconversion of the remaining sugar mixture. Each stage of such a two-stage process could itself be comprised of a microbial consortium, with members each carrying out a specific detoxification or conversion. The increasing interest and applications of microbial consortia have been reviewed [54].

Alkaline pre-treatment of biomass yields a hydrolysate stream-containing numerous aromatic acids such as vanillic acid, ferulic acid, and 4-hydroxybenzoic acid [22]. Although a few biochemical production strains tolerate significant quantities of these inhibitors, several strains of *Amycolatopsis*, *Pseudomonas putida*, *Acinetobacter baylyi*, and *Rhodococcus jostii* metabolize many low-molecular-weight compounds normally considered to be inhibitors [40]. These bacteria represent good hosts for engineering towards the removal of inhibitors. *A. baylyi* ADP1 additionally possesses a highly efficient system for natural transformation and chromosomal incorporation of exogenous DNA [10, 57]. This genetic malleability facilitates the generation of mutants with specific chromosomal changes and also the evolution of new enzymes for lignin valorization [51]. In ADP1, an engineered deletion of *gcd*, a gene-encoding glucose dehydrogenase, fails to metabolize glucose, xylose, or arabinose. Furthermore, this strain metabolizes acetate, formate, and 4-hydroxybenzoate (4HB) simultaneously in the absence of glucose, or, when glucose is present, it degrades acetate and formate [21]. The *gcd* mutant also grows aerobically on citrate in an enzymatic rice straw hydrolysate prior to anaerobic growth of *Clostridium butyricum* for the production of hydrogen gas [21].

In this study, we used a consortium of ADP1-derived strains to metabolize both benzoate and 4HB as a model detoxification step prior to the conversion of glucose to ethanol by *Kluyveromyces marxianus* [2]. Because the optimal growth and fermentation conditions for this yeast are unfavorable to bacterial growth (42 °C and a pH less than 5.5), *K. marxianus* is uniquely suited to a two-stage process that requires bacterial inactivation in the second stage.

## Results and discussion

### Selective consumption of aromatic compounds

**Fig. 1:**
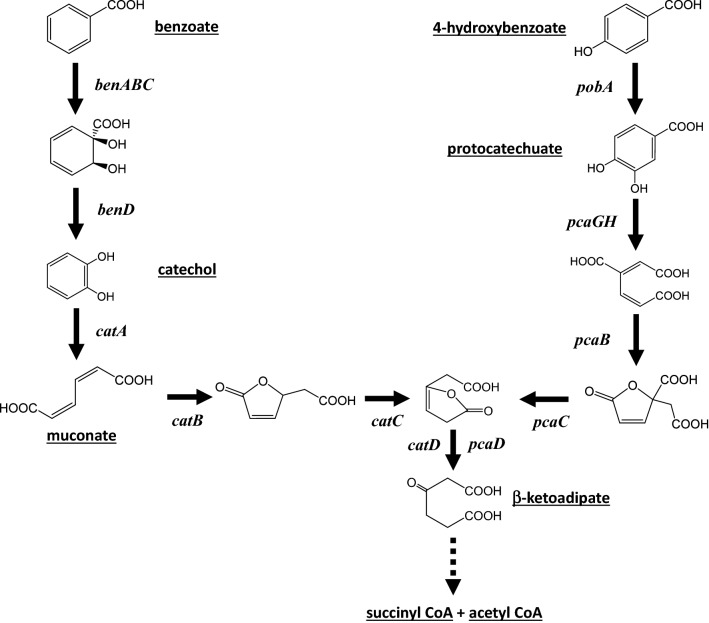
Catechol and protocatechuate branches (left and right, respectively) of the beta-ketoadipate pathway for the degradation of benzoate and 4HB by *A. baylyi* ADP1. Enzymes are indicated as gene products

*Acinetobacter baylyi* ADP1 consumes benzoate and 4HB by converting them to central metabolites (acetyl CoA and succinyl CoA) via the catechol and protocatechuate branches, respectively, of the β-ketoadipate pathway (Fig. 1). When both compounds are present, ADP1 metabolizes them sequentially: 4HB consumption commences only when most of the benzoate is depleted [4]. We hypothesized that both benzoate and 4HB could be simultaneously consumed by a consortium of two different ADP1-derived mutants, one of which would consume only benzoate and another that would consume only 4HB. The strain that consumes 4HB could be prevented from degrading benzoate by inactivating a gene needed for catechol formation. An analogous mutant blocked in the protocatechuate branch of the pathway could consume benzoate. More generally, a consortium of specifically engineered mutants might consume different aromatic compounds in a mixture regardless of the composition of the hydrolysate.

**Fig. 2:**
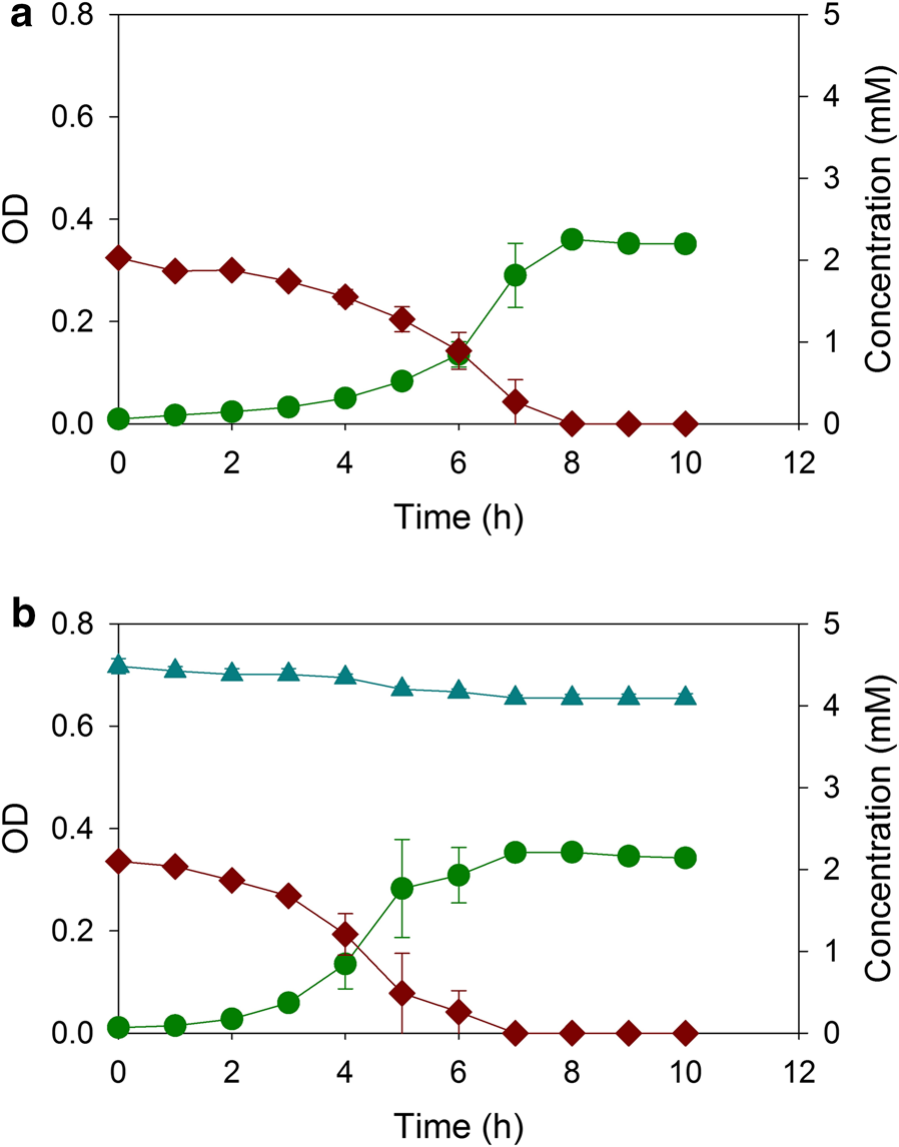
Growth of ACN462 on **a** benzoate and **b** benzoate and 4-hydroxybenzoate: OD (dark green circle), benzoate (brown diamond), and 4-hydroxybenzoate (blue up pointing triangle). Error bars represent standard error of the mean

**Fig. 3:**
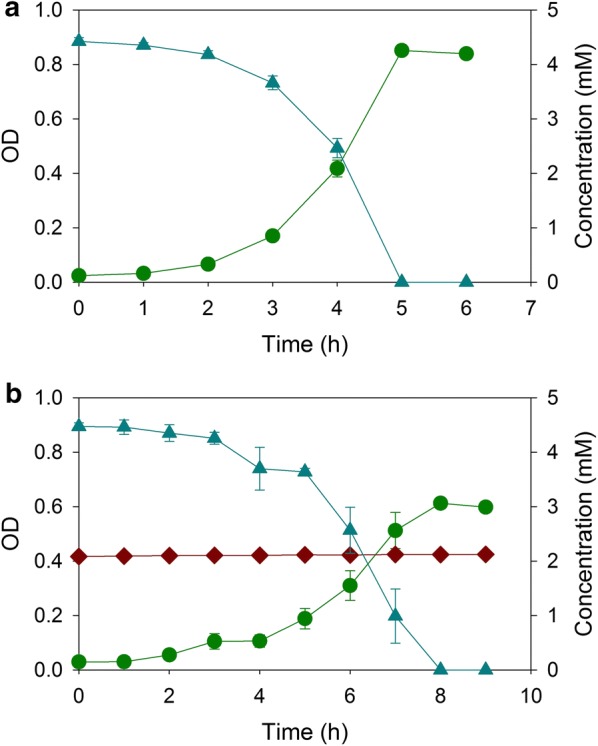
Growth of ACN472 on **a** 4-hydroxybenzoate and **b** benzoate and 4-hydroxybenzoate: OD (dark green circle), benzoate (brown diamond), 4-hydroxybenzoate (blue up pointing triangle). Error bars represent standard error of the mean

In an ADP1-derived mutant, ACN462, the deletion of the *pcaGH* genes, which encode protocatechuate 3,4-dioxygenase, prevented catabolism of protocatechuate and thereby also 4HB. ACN462 consumed 2 mM benzoate as the sole carbon source in less than 8 h yielding an OD of about 0.35 (Fig. 2a). When ACN462 was grown in the same medium that additionally contained 5 mM 4HB, there was no change in benzoate metabolism or cell yield, suggesting that 4HB had no inhibitory effect (Fig. 2b). A different ADP1-derived mutant, ACN472, was unable to consume benzoate because of the deletion of *benD*, which encodes the dehydrogenase needed for the conversion of benzoate to catechol. ACN472 depleted 5 mM 4HB as the sole carbon source in 5 h (Fig. 3a). When the same medium additionally contained 2 mM benzoate, ACN472 exclusively metabolized 4HB within 8 h (Fig. 3b). The deletion of the *pcaGH* and *benD* genes in separate ADP1-derived strains resulted in one strain that only consumed benzoate (ACN462) and one that only consumed 4HB (ACN472) when provided with both aromatic substrates.

### Selective growth in the presence of sugars

**Fig. 4:**
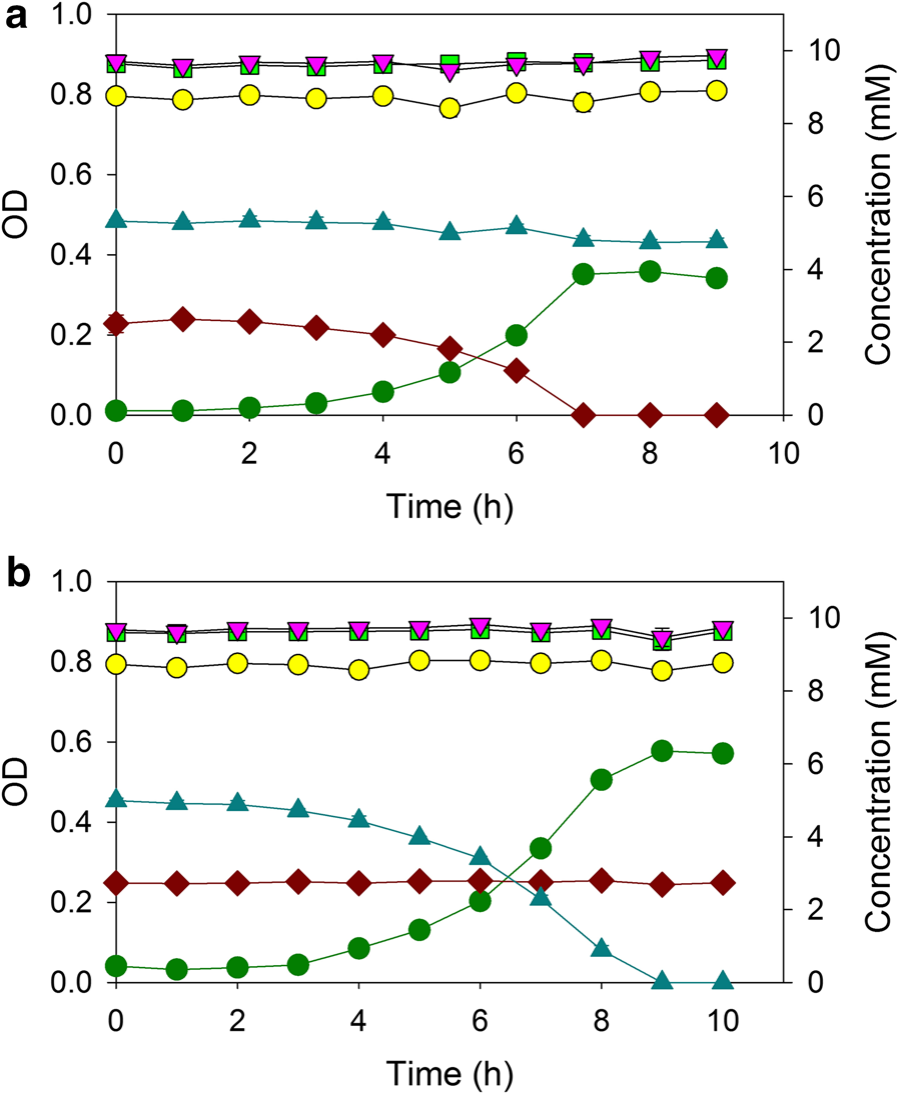
Growth of **a** ACN2090 and **b** ACN2091 on mixtures of benzoate, 4-hydroxybenzoate, glucose, xylose, and arabinose: OD (dark green circle), benzoate (brown diamond), 4-hydroxybenzoate (blue up pointing triangle), glucose (yellow circle), xylose (light green square), and arabinose (pink down pointing triangle). Error bars represent standard error of the mean

Real hydrolysates contain sugars, which, generally, are poor growth substrates for ADP1. This bacterium is unable to metabolize xylose or arabinose, and its utilization of glucose can be prevented by deletion of the *gcd* gene [21]. *A. baylyi* strains were next constructed to prevent the consumption of glucose during the selective degradation of aromatic compounds. One mutant, designated ACN2090, was engineered to carry two deletions, Δ*pcaGH* and Δ*gcd*. Another mutant, designated ACN2091, was engineered to carry Δ*benD* and Δ*gcd*. In a mixture of glucose, xylose, arabinose, 4HB, and benzoate, ACN2090 exhausted 2 mM benzoate within 7 h (Fig. 4a), and ACN2091 consumed 5 mM 4HB within 9 h (Fig. 4b). These two substrate-selective strains were largely unaffected by the presence of glucose, xylose, and arabinose.

**Fig. 5:**
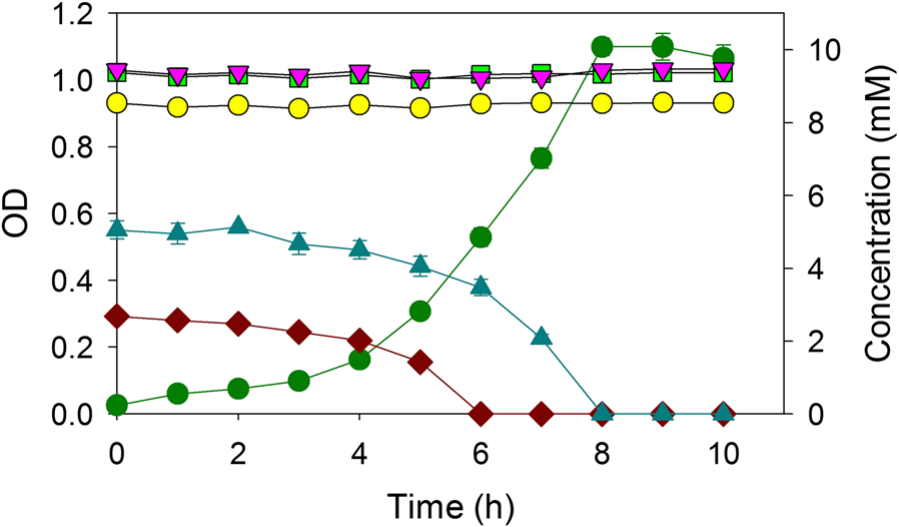
Growth of ACN2090 and ACN2091 together on a mixture of benzoate, 4-hydroxybenzoate, glucose, xylose, and arabinose: OD (dark green circle), benzoate (brown diamond), 4-hydroxybenzoate (blue up pointing triangle), glucose (yellow circle), xylose (light green square), and arabinose (pink down pointing triangle). Error bars represent standard error of the mean

We next tested metabolism by a consortium of both strains, ACN2090 and ACN2091. When provided with a mixture of sugars and aromatic compounds, this consortium simultaneously metabolized both benzoate and 4HB within 8 h (Fig. 5). In contrast to the fashion in which benzoate and 4HB are consumed by the wild-type strain (ADP1), we observed no delay in the commencement of 4HB degradation. Each strain in the presence of two carbon sources behaved the same as in the presence of the single carbon source (i.e., Fig. 2a, b).

### Growth in continuous culture

The studies described so far were completed under batch conditions in which the bacteria initially experience an excess of the carbon source(s). We sought to compare these results using a batch culture to the behavior of strains in a chemostat whereby a nutrient feed is supplied continuously at a constant rate, and an effluent from the bioreactor is withdrawn at the same rate resulting in a constant volume bioreactor. At the initiation of such a process, cell density increases until one (limiting) nutrient is depleted, and a steady-state is achieved. If the carbon sources are limiting, then the steady-state concentrations of those substrates would be very low throughout the process. Under these conditions, cells often are able to consume multiple substrates simultaneously and avoid the diauxie that is observed during batch growth [13]. Moreover, changes in the feed composition of the limiting nutrients should affect only the cell density and not the effluent concentration of the limiting nutrients (as long as they remain limiting). Thus, a variable composition but constant-flowrate feed introduced into a “chemostat” would tend to maintain a single effluent composition. As a general practice, such a process would not only generate a consistent effluent for a subsequent process, but it can also maintain that stability when confronted with a variable composition feed such as those that might be encountered in lignocellulosic hydrolysates.

**Fig. 6:**
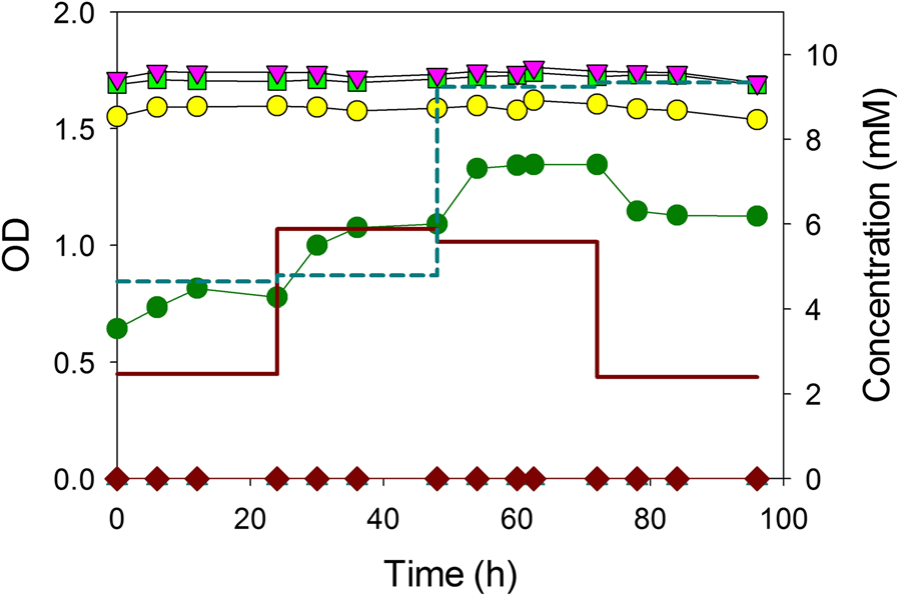
Growth of ACN2069 in continuous chemostat culture: OD (dark green circle), benzoate (brown diamond), 4-hydroxybenzoate (blue up pointing triangle), glucose (yellow circle), xylose (light green square), and arabinose (pink down pointing triangle). The feed concentrations (each) of glucose, xylose, and arabinose were maintained at nominally 10 mM, and the concentrations of benzoate (brown straight line) and 4-hydroxybenzoate (blue dashed lines) were step-changed approximately every 24 h as described in “Materials and methods”

Under carbon limitation, we expected *A. baylyi* to consume benzoate and 4HB simultaneously. To prevent glucose catabolism in an otherwise wild-type strain, we constructed ACN2069 (Δ*gcd*). This single strain could then be used to consume mixtures of aromatic compounds and sugars. ACN2069 was grown in a chemostat to degrade both benzoate and 4HB using a constant dilution rate of 0.2 h^−1^. To examine the robustness of this continuous process, the feed composition (but not flowrate) was shifted every 24 h: at the onset, the nominal composition was 2 mM benzoate and 5 mM 4HB; at 24 h benzoate shifted to 5 mM; at 48 h, the feed contained 10 mM 4HB; and then, finally at 72 h, the feed shifted to 2 mM benzoate. Throughout the process, the influent concentration of sugars remained nominally 10 mM glucose, 10 mM xylose, and 10 mM arabinose. The system was allowed about five residence times during each feed, so that a steady-state would essentially be achieved for that feed composition. Figure 6 shows the results of this 96 h process. The system maintained a negligible effluent concentration of benzoate and 4HB, despite the shifting feed composition. The cell density increased or decreased in proportion to the total quantity of metabolizable carbon/energy sources. During the process, the concentration of the three sugars remained at 10 mM, demonstrating that they were not consumed over the course of the 4 days, and that glucose-degrading mutants did not arise spontaneously [5]. Since the strains never encountered high and potentially toxic concentrations of the aromatic substrates, such a continuous process using substrate-selective microbes would be a very effective means to remove a portion of the carbon sources from a variable feed stream.

### Removal of inhibitors and formation of a biochemical product

**Fig. 7:**
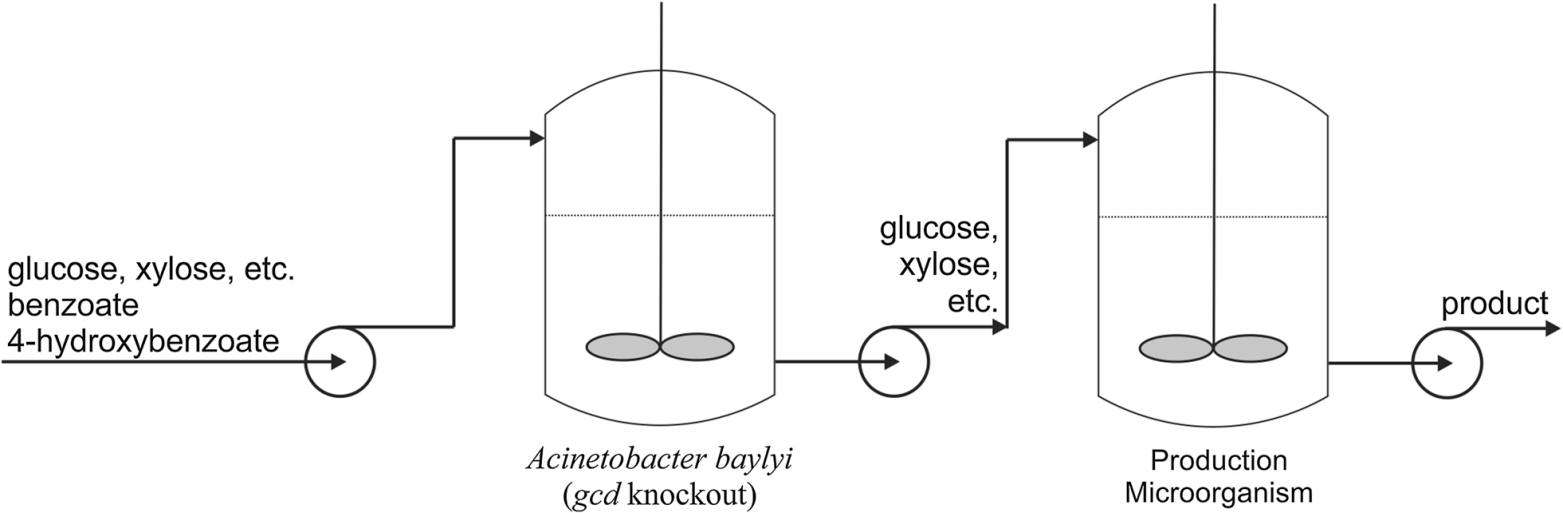
Two bioreactor process for continuous removal of aromatic inhibitors and production of any growth-associated product. Reactor 1 (left) allows for continuous growth of *Acinetobacter baylyi* on mixtures of aromatics such as benzoate and 4-hydroxybenzoate. A strain with a *gcd* knockout prevents glucose, xylose, and arabinose consumption. An industrial microbe may now grow continuously in Reactor 2 (right) on the residual sugars to generate a biochemical product. Conditions found in Reactor 2 (e.g., different temperature or pH) should prevent metabolism of the product by *A. baylyi*

The results suggest that a two-stage microbial process could be developed to (1) detoxify an inhibitor-containing feed stream and (2) convert the resulting sugar stream into a biochemical product. Such a process might employ any of several operational modes. For example, a consortium of inhibitor-consuming microbes could remove multiple components during a batch culture, with each microbe simultaneously degrading one target compound (i.e., Fig. 5). Subsequently, after the depletion of inhibitors, one or more microbes could be introduced into the same batch to convert the remaining sugars into a desired biochemical product, such as ethanol. Alternatively, a continuous process could be developed whereby the inhibitors are removed in one bioreactor, and in a separate, second reactor the sugars are converted into a product. Each stage of such a two-stage process could involve a different microbial species and different environmental conditions. An example of an envisioned continuous process is shown in Fig. 7, where appropriate strain(s) are introduced into a second reactor to convert the sugar mixture into a product. In each reactor of this “fully continuous” configuration, the microbes must be growing, and thus, the product must be a growth-associated product such as pyruvate [26]. One critical aspect of implementing a two-stage detoxification/conversion process using any operational mode is that the first inhibitor-consuming microbe(s) must be unable to metabolize the product under the environmental conditions found in the second stage. This goal could be accomplished by deleting catabolic routes in the strain(s) used for detoxification, or by operating the second stage in an environment unfavorable to the growth of the first-stage detoxification microbes. An advantage of such a two-stage process, in contrast to simultaneous degradation and conversion, is that the environmental conditions for each stage (medium, temperature, pH, etc.) can be independently optimized.

To demonstrate a two-stage biological process to detoxify a stream and generate a product, we set up the first stage as a batch process to remove benzoate and 4HB using two strains of *A. baylyi.* For the second production stage, *Kluyveromyces marxianus* was then used in the same reactor to convert the remaining sugar, glucose, into ethanol. Like many other microbes, we determined that the growth of *K. marxianus* was inhibited by 2.5 mM benzoate (data not shown). Although ADP1 is able to metabolize ethanol [46], *K. marxianus* grows at 42 °C and a pH of 5.0–5.5 [24, 37], conditions at which ADP1 will neither grow nor metabolize ethanol. After 4HB and benzoate in the initial batch were depleted, a more concentrated mixture of 4HB and benzoate was slowly introduced. After the degradation of the inhibitors, the temperature was increased and the pH lowered to eliminate active *A. baylyi* cells. Before the introduction of the yeast, the temperature was adjusted to operating conditions suitable for the yeast (42 °C and pH of 5.2).

**Fig. 8:**
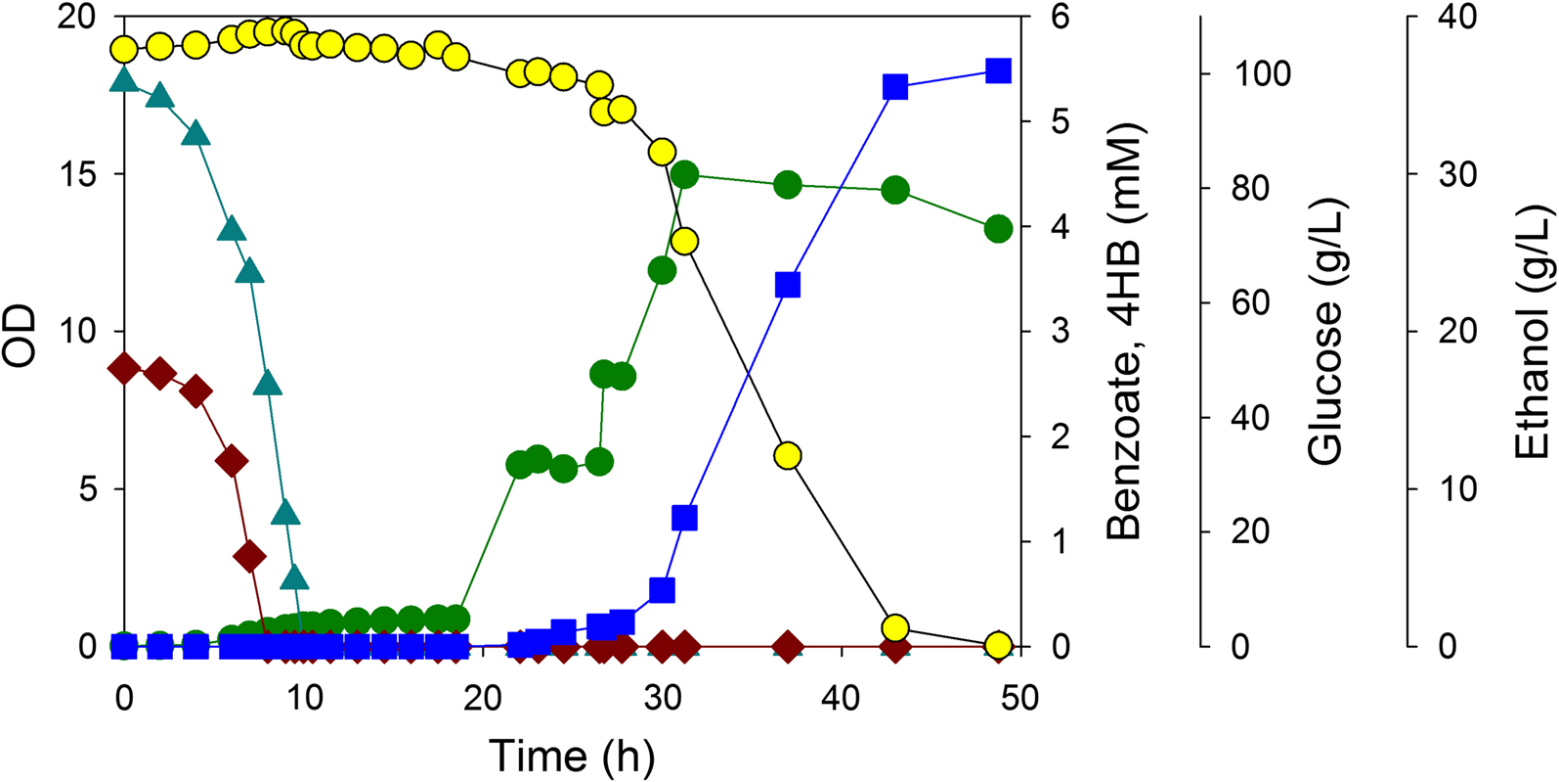
Two-stage process for the degradation of benzoate (brown diamond) and 4HB (blue up pointing triangle) in a mixture with glucose (yellow circle) and the subsequent conversion to ethanol (blue square). The total bacterial + yeast OD (dark green circle). ACN2090 and ACN2091 were inoculated into a mixture of 100 g/L glucose, 5 mM 4HB, and 2.5 mM benzoate. After 9.7 h, both aromatic carbon sources were depleted, and a feed of 100 g/L glucose, 11 mM 4HB, and 6 mM benzoate was introduced at a constant flowrate of 1 mL/min (an initial dilution rate of 0.06 h^−1^), resulting in an increase of the bioreactor volume to approximately 1.6 L after an additional 9.8 h. After this feed was terminated, the temperature was raised to 55 °C and the pH lowered to 4.3, and then, the temperature quickly lowered to 42 °C and the pH increased to 5.2 (the complete cycle required 3.5 h). After inoculation with *K. marxianus,* aerobic conditions were provided for 9.3 h before switching to anaerobic conditions for the final 17.5 h

The 1.0 L batch process began with a reactor containing nominally 100 g/L glucose, 5 mM 4HB, and 2.5 mM benzoate (results are shown in Fig. 8). After 9.7 h, both aromatic carbon sources were depleted by ACN2090 and ACN2091, and a feed of 100 g/L glucose, 11 mM 4HB, and 6 mM benzoate was introduced at a constant flowrate of 1 mL/min (corresponding to an initial dilution rate of 0.06 h^−1^), resulting in an increase of the bioreactor volume to approximately 1.6 L after an additional 9.8 h. The feed was terminated, the temperature was raised to 55 °C and the pH lowered to 4.3, and finally, the temperature was quickly lowered to 42 °C and the pH increased to 5.2 (the heating, holding and cooling cycle required 3.5 h). After inoculation with *K. marxianus,* aerobic conditions were provided for 9.3 h before switching to anaerobic conditions for the final 17.5 h. Similar to shake flask studies using a microbial consortium (Fig. 5), the initial 4HB and benzoate were consumed simultaneously (Fig. 8). Although the feed contained 4HB and benzoate at over twice the concentration present at the onset of the batch phase, the system maintained a zero concentration of aromatics during that portion of the process. This result demonstrates benefits of a continuous process: the presence of active cells during the feeding period, and the dilution effect achieved by introducing a concentrated feed into a larger dilute reservoir, result in a negligible concentration of the inhibitors. During the anaerobic phase (21.3 h–49 h), 36.6 g/L ethanol was generated at a yield of 0.38 g/g glucose (Fig. 8).

In this study, we described approaches (using a single strain or two substrate-selective strains, as necessary) to remove two aromatic hydrocarbons simultaneously from a simulated lignocellulosic hydrolysate. We also demonstrated the degradation of two aromatic hydrocarbons followed by the conversion of a residual sugar into ethanol. In this case, a two-stage batch process was used, because the product, ethanol, is a non-growth-associated product. A continuous process allowing for high volumetric productivity would be advantageous for growth-associated products, in which case the dilution rates of each stage (and vessel) can be independently established. Conceptually, the process can be extended to other inhibitors or sugars found in real hydrolysates. That is, additional strains which degrade components of lignocellulosic hydrolysate can be made substrate-selective and introduced into the complex mixture found in a hydrolysate.

The proposed microbial consortium process provides another approach to the wide range of biological, physical, and chemical methods studied for the detoxification of lignocellulosic hydrolysates [29, 30]. In addition to the selective removal of specific compounds described in this study, biological methods for addressing inhibition include the use of enzyme laccase and peroxidase enzymes [18] or fungi directly [33, 59], the use of a co-culture of detoxifying and fermenting microbes [58], and the adaptation of strains to hydrolysates [42, 44]. More prevalent are physical and chemical methods including over-liming [27], adsorption on activated charcoal [35] or ion-exchange [34]. The use of a consortium of substrate-selective microbes has the advantages of being well aligned with the equipment and culture requirements of a subsequent bioconversion step, and being amenable to the variability of the hydrolysate.

## Materials and methods

### Strains and plasmids

Bacterial strains were derived from *A. baylyi* ADP1 (Table 1). Chromosomal mutations were engineered via allelic replacement with linearized plasmids that transformed naturally competent *A. baylyi* hosts, as previously described [32]. Each new strain is a transformant in which a plasmid-borne allele replaced the corresponding chromosomal locus. The genotypes of new strains were confirmed by PCR and regional DNA sequencing.

**Table 1:** *Acinetobacter baylyi* strains used in this study

Strain	Relevant characteristics	Sources
ADP1	Wild type (BD413)	Juni and Janik [19]
ACN462	Δ*pcaHG5462* Deletion of DNA from *1,717,153* to *1,715,814*^a^ replaced with GCGGCCGC, the recognition sequence for *Not*I PCR fragment X ADP1; screened for loss of ability to grow on 4HB ^c^	[7]
ACN472	Δ*benD5472* Deletion of DNA from *1,437,252* to *1,438,007*^a^ replaced with GCGGCCGC, the recognition sequence for *Not*I PCR fragment X ADP1; screened for loss of ability to grow on benzoate ^b^	Eby [7]
ACN2069	Δ*gcd*::*sacB*-Km^R^*52069* pBAC1566/AatII X ADP1; selected by Km ^R^	This study
ACN2070	Δ*gcd*::*sacB*-Km^R^*52069*; Δ*pcaHG5462* pBAC1566/AatII X ACN462; selected by Km ^R^	This study
ACN2071	Δ*gcd*::*sacB*-Km^R^*52069*; Δ*benD5472* pBAC1566/AatII X ACN472; selected by Km ^R^	This study
ACN2089	Δ*gcd52089* pBAC1565/AatII X ACN2069; selected by growth in the presence of sucrose ^b^	This study
ACN2090	Δ*gcd52089*; Δ*pcaHG5462* pBAC1565/AatII X ACN2070; selected by growth in the presence of sucrose ^b^	This study
ACN2091	Δ*gcd52089*; Δ*benD5472* pBAC1565/AatII X ACN2071; selected by growth in the presence of sucrose ^b^	This study

Strains were derived from ADP1, previously known as *Acinetobacter calcoaceticus* or *Acinetobacter* sp. [53]

^a^Italics numbers correspond to positions on the ADP1 chromosome in NCBI entry NC_005966

^b^Underlined text indicates the donor DNA and, where relevant, the restriction enzyme used to linearize a plasmid (pBAC number/enzyme). The donor DNA was used to transform (X) the indicated recipient strain

Plasmids are shown in Table 2. To construct strains unable to degrade glucose, two plasmids were made with a defined deletion of *gcd* (locus designation ACIAD_RS13470, or ACIAD2983, at position 2,911,967 ← 2,914,372, in the ADP1 genome, NC_005966 in NCBI). One of these plasmids, pBAC1565, carries ADP1 DNA surrounding *gcd* from genomic positions 2,909,747 (downstream of *gcd*) to 2,916,396 (upstream of *gcd*). In this region, the coding sequence of *gcd* was deleted (except for the translational start and stop signals). The deletion corresponds to genome positions 2,911,970–2,914,369. In place of the deleted DNA, a *Bam*HI recognition sequence (GGATCC) was inserted. This plasmid was constructed by joining two PCR products using splicing by overlap extension PCR (SOEing) [15]. One PCR product was generated with primers SRB36 and SRB38 and the other with SRB39 and SRB37 (Additional file 1: Table S1). The joined SOEing fragment (made with primers SRB39 and SRB38) was ligated into the cloning vector pUC18 after restriction digestion with *Pst*I and *Eco*53KI. The second plasmid carrying a *gcd* deletion, pBAC1566, was created to carry a selectable/counter-selectable marker inserted into the engineered BamHI site of pBAC1565, within the deleted *gcd* region. This marker, a *sacB*-Km^R^ cassette, was excised from pRMJ1 [17].

**Table 2:** Plasmids used in this study

Plasmid	Relevant characteristics	Source
pUC18	Ap^R^; cloning vector	Yanisch-Perron et al. [56]
pRMJ1	Source of *sacB*-Km^R^ cassette	Jones and Williams [17]
pCR2.1-TOPO	Vector for cloning PCR products; Ap^R^, Km^R^	ThermoFisher (Invitrogen)
pBAC890	Engineered allele: Δ*benD5472* PCR fragment with this allele was constructed by SOEing (see “Materials and methods”). This fragment was cloned into pCR2.1-TOPO	This study
pBAC1565	Engineered allele: Δ*gcd52089* Carries ADP1 DNA upstream and downstream of the deleted *gcd* in pUC18; ADP1 region corresponds to genome positions *2,909,747*–*2,916,396*^a^ with a deletion of *2,911,970* to *2,914,369*^a^. The deleted DNA is replaced by a *Bam*HI recognition sequence (GGATCC)	This study
pBAC1566	Engineered allele: Δ*gcd*::*sacB*-Km^R^*52069* The *sacB*-Km^R^ cassette, excised from pRMJ1 as a *Bam*HI fragment was inserted in the corresponding restriction site of pBAC1565	This study

^a^Italics numbers correspond to positions on the ADP1 chromosome in NCBI entry NC_005966

Three strains were constructed in which *gcd* was deleted and replaced with the *sacB*-Km^R^ cassette (allele Δ*gcd*::*sacB*-Km^R^*52069*): ACN2069, ACN2070, and ACN2071. These strains were selected by resistance to kanamycin (Km) after linearized pBAC1566 transformed three recipient strains, ADP1, ACN462, and ACN472, respectively. Unmarked deletion strains, ACN2089, ACN2090, and ACN2091, were generated using strains that carry *sacB* as recipients for transformation by linearized pBAC1565. Strains in which the unmarked *gcd* deletion (allele Δ*gcd52089*) replaced the corresponding region of the recipient strain were isolated in the presence of sucrose (5% w/v at 30 °C) to select for the loss of *sacB*.

Two strains with deletions affecting aromatic compound catabolism were previously constructed, ACN462 [7] and ACN472. To generate these strains, an SOEing PCR product was used to transform ADP1. Transformants corresponding to ACN462 and ACN472 were initially identified after screening for the loss of growth on compounds degraded via protocatechuate or benzoate, respectively. The PCR fragment used to generate ACN472 was an SOEing product made with two primers (BenD-Up and BenD-LT) that joined two PCR fragments, one made using BenD-Up with BenD-Del2 and the other made using BenD-Del1 with BenD-LT. A plasmid (pBAC890) that carries DNA of this SOEing product, which has the Δ*benD5472* allele, was generated using a TOPO TA cloning kit (ThermoFisher Scientific, Waltham, MA USA).

*Kluyveromyces marxianus* ATCC 26548 was used without modification. The yeast was routinely grown in YPD medium at 42 °C and pH of 5.5 containing 20 g/L glucose, 20 g/L peptone, and 10 g/L yeast extract.

### Media and growth conditions

Defined ADP1 medium was adapted from Shanley et al. [43] and contained (per liter): 3.35 g Na_2_HPO_4_·7H_2_O, 1.70 g KH_2_PO_4_, 1.00 g (NH_4_)_2_SO_4_, 59 mg MgSO_4_·7H_2_O, 6.7 mg CaCl_2_·2H_2_O, 2.2 mg ZnSO_4_·7H_2_O, 1.4 mg FeSO_4_·7H_2_O, 0.31 mg MnSO_4_·H_2_O, 78 μg CuSO_4_·5H_2_O, 50 μg Co(NO_3_)_2_·6H_2_O, 35 μg Na_2_B_4_O_7_·10H_2_O, 19 μg (NH_4_)_6_Mo_7_O_24_·4H_2_O, 0.5 mg EDTA, and 20 mg nitrilotriacetic acid, and the carbon source sterilized separately. This medium was supplemented with benzoate, 4HB, and/or sugars as described.

### Shake flask studies

In a single strain experiment, cells were first grown in 25 mL ADP1 medium in 125 mL baffled shake flasks, and after 24 h, 4 mL was used to inoculate each of triplicate 500 mL baffled shake flask-containing 100 mL ADP1 medium, from which measurements were made. In an experiment using two strains (i.e., a consortium), each strain was first grown separately in 25 mL ADP1 medium as before, and then, 2 mL from each flask used as inoculum for triplicate 500 mL shake flasks with 100 mL ADP1 medium. All flasks nominally contained 2 mM benzoate and 5 mM 4HB and were incubated at 37 °C and 250 rpm (19 mm pitch). The pH of the medium was 7.0.

### Chemostat process

Continuous degradation of benzoate and 4HB in the presence of sugars was studied in the chemostat mode at a dilution rate of 0.20 h^−1^. ACN2069 was first grown in 25 mL ADP1 medium in 125 mL shake flasks as described above and after 24 h transferred to a 2.5 L bioreactor (Bioflo 2000, New Brunswick Scientific Co., New Brunswick, NJ, USA) containing 1.0 L ADP1 medium. During the process, four different carbon source compositions were supplied using ADP1 medium: From 0 to 24 h: 2 mM benzoate/5 mM 4HB/10 mM sugars (i.e., 10 mM glucose, 10 mM xylose, and 10 mM arabinose); from 24 to 48 h: 5 mM benzoate/5 mM 4HB/10 mM sugars; from 48 to 72 h: 5 mM benzoate/10 mM 4HB/10 mM sugars; from 72 to 96 h: 2 mM benzoate/10 mM 4HB/10 mM sugars.

### Two-stage bacteria–yeast process

A two-stage biological process was used to detoxify a stream and generate ethanol. In a 2.5 L vessel containing 1.0 L of nominally 2 mM benzoate/5 mM 4HB and 100 g/L glucose in ADP1 medium was inoculated with 25 mL of ACN2090 and 25 mL of ACN2091 grown in 125 mL baffled flasks (as described above). After both aromatic hydrocarbons were depleted, a solution containing 5 mM benzoate/10 mM 4HB and 100 g/L glucose in ADP1 medium was introduced at 1 mL/min for 9 h. Then, the pH was decreased to 4.3 with sulfuric acid, and over the course of 2 h, the temperature was increased to 55 °C, held for about 1 h, and then decreased to 42 °C over the course of 0.1 h. After the pH was increased to 5.2, approximately 2 g (dry cell basis) of *K. marxianus* in 35 mL ADP1 medium was used to inoculate the fermenter. After inoculation, air was sparged at 1 L/min for 9.3 h before switching to anaerobic conditions (sparging N_2_ at 0.1 L/min) for the final 17.5 h.

### Analytical methods

The optical density at 600 nm (OD) (UV-650 spectrophotometer, Beckman Instruments, San Jose, CA, USA) was used to monitor cell growth. Liquid chromatography using a Coregel 64-H ion-exclusion column (Transgenomic Ltd., Glasgow, United Kingdom) with a mobile phase of 4 mN H_2_SO_4_ and RI detection was used to quantify all extracellular organic compounds [8].

## Additional file

**Additional file 1: Table S1.** Primers used in this study.
