# Supplementary material for: Removal of aromatic inhibitors produced from lignocellulosic hydrolysates by Acinetobacter baylyi ADP1 with formation of ethanol by Kluyveromyces marxianus
Source: Biotechnol Biofuels. 2019 Apr 23;12:91. doi: 10.1186/s13068-019-1434-7 (PMC6477725; doi:10.1186/s13068-019-1434-7)
Supplement: Supplementary file 1 — Additional file 1: Table S1. Primers used in this study. [file 13068_2019_1434_MOESM1_ESM.docx]

Removal of aromatic inhibitors produced from lignocellulosic hydrolysates by *Acinetobacter baylyi* ADP1 with formation of ethanol by *Kluyveromyces marxianus*

Anita Singh et al. Additional Information

**Table S1.** Primers used in this study.

| Primer | Sequence (5' 🡪 3') | Notes |
| --- | --- | --- |
| SRB36 | ggtggtctcatg**GGATCC**taatcattgttattattcaccataaaaaaa | Used in SOEing reaction (paired with SRB38) to introduce BamHI site (bold) in place of *gcd* in pBAC1565 |
| SRB37 | gtgaataataacaatgatta**GGATCC**catgagaccacctcgaataat | Used in SOEing reaction (paired with SRB39) to introduce BamHI site (bold) in place of *gcd* in pBAC1565 |
| SRB38 | tttaagcccg**ctgcag**cttcctgaatgtc | Used as outside primer to join PCR fragments by SOEing. Used to introduce PstI site (bold) for cloning DNA into pUC18 to make pBAC1565 |
| SRB39 | AATCA**GAGCTC**CAAGTTTGGCAGCAGATGT | Used as outside primer to join PCR fragments by SOEing. Used to introduce Eco53KI site (bold) for cloning DNA into pUC18 to make pBAC1565 |
| SRB40 | gcttcaattggcgacatcttg | Used as sequencing primer |
| SRB41 | cttgatcctgacgtaaatccagataagt | Used as sequencing primer |
| SRB42 | aattttggatgctcggcaaacac | Used as sequencing primer |
| SRB43 | tgtgcataaccacggtcatattct | Used as sequencing primer |
| SRB44 | gcgatggacacgatattaagaataaagc | Used as sequencing primer |
| SRB45 | gaactcgcctcttgccaaag | Used as sequencing primer |
| SRB46 | tggaacgtgaaaatttgattgggc | Used as sequencing primer |
| BenD-Del1 | aacgttttgaacataaa**gcggccgc**tctgatggtcatta | Used in SOEing PCR (with BenD-LT) to introduce NotI site (bold) into deleted *benD* region |
| BenD-Del2 | TAAATAATGACCATCAGA**GCGGCCGC**TTTATGTTCAAAA | Used in SOEing PCR (with BenD-Up) to introduce NotI site (bold) into deleted *benD* region |
| BenD-Up | GGCACATGCAGAAAGTCAACA | Used as end primer to join PCR fragments by splicing to engineer the Δ*benD5472* allele |
| BenD-LT | CGGCACCGATCGAAATAGC | Used as end primer to join PCR fragments by splicing to engineer the Δ*benD5472* allele |
